# How depression affects school social adaptation: the mediating role of sleep quality and the buffering effect of physical activity

**DOI:** 10.3389/fspor.2025.1716670

**Published:** 2025-11-20

**Authors:** Chuan Chen, Kaihua Liu, Zhidong Zhou

**Affiliations:** School of Sports Science, Jishou University, Jishou, China

**Keywords:** depression, sleep quality, school social adaptation, physical activity, moderated mediation model, adolescents

## Abstract

**Background and objectives:**

The negative impact of depression on adolescents’ school social adaptation has been widely confirmed. However, the underlying mechanisms, particularly the synergistic roles of sleep quality and physical activity, remain inadequately explored. This study aims to construct a moderated mediation model to examine the mediating effect of sleep quality between depression and school social adaptation, as well as the moderating effect of physical activity in this pathway.

**Methods:**

A cross-sectional survey design was employed, with 2,354 adolescents (1,098 male, 1,256 female) from six provinces in China participating via convenience sampling. The Patient Health Questionnaire (PHQ-9), Pittsburgh Sleep Quality Index (PSQI), School Belonging Scale (SBS), and Physical Activity Rating Scale (PARS-3) were used to assess depression, sleep quality, school social adaptation, and physical activity, respectively. Mediation and moderation effects were tested using the SPSS PROCESS macro.

**Results:**

Correlation analysis revealed a significant negative correlation between depression and school social adaptation (*r* = −0.410, *p* < 0.001). Mediation effect testing indicated that sleep quality partially mediated the relationship between depression and school social adaptation. Depression not only directly predicted school social adaptation negatively (*β* = −0.430, *p* < 0.001) but also indirectly weakened school social adaptation through its impact on sleep quality (*β* = −0.294, *p* < 0.001), which, in turn, positively predicted school social adaptation (*β* = 0.261, *p* < 0.001). Moderated mediation analysis further revealed that physical activity significantly moderated both the first half of the “depression → sleep quality” pathway and the direct path from depression to school social adaptation, suggesting that higher levels of physical activity effectively buffer the negative effects of depression on sleep quality and school social adaptation.

**Conclusions:**

Sleep quality is a crucial mediating mechanism through which depression affects school social adaptation, while physical activity plays a positive moderating role in this process. The findings suggest that encouraging adolescents to engage in regular physical activity may serve as an effective intervention strategy to improve sleep quality, alleviate depressive symptoms, and ultimately enhance their school social adaptation. As a primary goal, the abstract should render the general significance and conceptual advance of the work clearly accessible to a broad readership. References should not be cited in the abstract. Leave the Abstract empty if your article does not require one—please see the “Article types” on every Frontiers journal page for full details.

## 1 Introduction

In the era of globalization and knowledge-based economy, the mental health and social functioning of adolescents have become key issues in education and public policy. In recent years, Chinese adolescents have been subjected to significant academic and competitive pressures due to the “one-exam-determines-all” nature of the Zhongkao (high school entrance exam) and Gaokao (college entrance exam), especially during crucial stages of educational advancement. The immense academic pressure often leads adolescents to proactively or passively “sacrifice” sleep time in exchange for study time, which in turn impairs their emotional stability and psychological regulation abilities (1). Although the “Double Reduction” policy, which aims to alleviate extracurricular academic burdens, has been fully implemented, there are still instances in some regions and stages of execution where academic burdens are subtly increased through mechanisms such as “after-school tutoring”. This has prevented the policy from fully achieving its intended stress-reducing effect, leaving academic pressure and associated depressive symptoms widespread among students (2). In this “academically-driven” cultural environment, adolescents' sleep time and opportunities for physical activity are structurally squeezed, posing unique and complex challenges to their mental health and social adaptation. These interrelated factors significantly increase the risk of depression and often weaken social adaptability by affecting sleep quality.Depression is characterized by persistent low mood, anhedonia, and reduced motivation, which frequently lead students to withdraw from classes, experience social withdrawal, and weaken their support networks (3). Sleep disorders, including insomnia, fragmented sleep, and disrupted sleep structure, are common among depressed adolescents. These disorders contribute to impaired emotional regulation and cognitive function, serving as a key mechanism linking depression with school adaptation difficulties (4). Physical activity, as a modifiable health behavior, plays a protective role by improving sleep quality and alleviating depressive symptoms (5). Additionally, it fosters positive emotions, enhances self-efficacy, and cultivates a sense of belonging, thereby promoting better social adaptation (6). According to Self-Determination Theory, physical activity satisfies psychological needs for autonomy, competence, and relatedness, and may buffer the negative impact of depression on social functioning through improvements in sleep quality (7). Despite these insights, there is still a lack of integrated empirical research within the Chinese adolescent population to explore how depression, sleep quality, and physical activity jointly influence school social adaptation. This study aims to fill this gap by constructing a moderated mediation model based on Self-Determination Theory to examine the role of physical activity in the pathway from depression to school adaptation.

In the psychological development and socialization process of adolescents, school social adaptation is a crucial indicator of their mental health and social functioning. School social adaptation is typically defined as an individual's ability to actively cope with academic pressure, interpersonal relationships, and behavioral norms in the specific social environment of school, effectively participate in school life, establish positive interpersonal interactions, and achieve psychological satisfaction and a sense of accomplishment (8). In the context of China's collectivist cultural background, school social adaptation carries deeper significance. An individual's integration into the group, maintenance of interpersonal harmony, and adherence to collective norms are regarded as important social competencies. Therefore, when adolescents experience maladjustment, it not only affects their personal development but, in an environment that emphasizes “group conformity”, may also more easily trigger negative peer evaluations and exclusion. This, in turn, exacerbates their psychological stress and social withdrawal, creating a vicious cycle (9). According to Self-Worth Theory, when individuals receive positive feedback in social interactions, they develop positive self-evaluations, which, in turn, promote emotional regulation and psychological well-being (10). Thus, good school adaptation is not only a result of psychological development but also a positive cycle that is continuously reinforced after the fulfillment of basic needs. Research has shown that well-adapted students tend to have higher learning motivation and classroom participation, demonstrating greater psychological resilience and problem-solving abilities when facing academic challenges, which leads to better academic performance (11). In terms of interpersonal relationships, they are more likely to build trust and cooperation with teachers and peers, gain a sense of belonging and emotional support, and develop positive social cognition and prosocial behaviors (12). Furthermore, they typically possess higher self-esteem, self-worth, and emotional stability, laying a solid foundation for their mental health and future development (13). However, the issue of poor school social adaptation is prevalent among adolescents and has far-reaching consequences. A study in China found that approximately 16.6% of adolescents experience significant difficulties in school adaptation, manifesting as low academic motivation, frequent interpersonal conflicts, increased disciplinary behavior, and poor emotional regulation (14). The situation in other countries is equally concerning. For example, a survey in Iceland revealed that nearly 50% of adolescents feel peer rejection, and over 90% of parents believe their children face peer adaptation issues (15). This phenomenon is not only an individual problem but also a widespread developmental challenge faced by adolescents in the context of changing social environments. Poor school adaptation has negative impacts on multiple levels. The most direct consequence is a decline in academic performance, with students often exhibiting behaviors such as truancy, school aversion, and consistently low academic outcomes (16). Moreover, difficulties in school adaptation can lead to isolation, frequent conflicts, reduced social support, and increased feelings of loneliness and helplessness in interpersonal relationships (17). Additionally, poor school adaptation is closely linked to high-risk behaviors, such as smoking, alcohol consumption, and even violent tendencies (18). Existing studies have indicated that adolescents with poor adaptation are 1.78 times more likely to smoke compared to their well-adapted peers (19). Furthermore, research has found that poor school adaptation is strongly associated with psychological disorders, such as autism and self-harm behaviors. Long-term struggles may also affect their future social functioning in areas like interpersonal relationships and career development (20). From a sociological perspective, poor school adaptation can be explained through Social Control Theory and Social Bond Theory. Durkheim (1895) argued that strong social bonds help maintain social order, whereas weak connections are more likely to lead to deviant behavior (21). Hirschi (1969) suggested that when individuals break their bonds with society and lack identification with rules and support from significant others, they are more likely to engage in deviant behavior (22). As an essential site for adolescent socialization, schools play a crucial role in adolescent adaptation through both structural support and emotional connections. When students lack a sense of belonging and identity, their adaptation difficulties often evolve into broader psychological and social issues.

The high prevalence and harmful effects of depressive symptoms in adolescents cannot be overlooked. Depression not only profoundly impacts an individual's emotional experiences and cognitive functions but may also serve as a significant risk factor hindering normal social adaptation. Depression is a mental disorder characterized by persistent low mood and a loss of interest or pleasure in daily activities, typically severe enough to affect an individual's social functioning and quality of life. Patients often describe their experience as “lacking the motivation to face each day” or “feeling sad and lonely for no reason”. The typical symptoms of depression include, but are not limited to, irritability, extreme fatigue, emotional numbness, and persistent sadness (23, 24). Currently, the detection rate of depressive symptoms in adolescents is alarmingly high. A systematic review and meta-analysis of 439 studies in China found the overall prevalence of depressive symptoms in adolescents to be 26.17% (25); global data also indicate that approximately 34% of adolescents aged 10 to 19 are at clinical risk for depression, with symptom severity worsening in regions such as the Middle East, Africa, and Asia (26). Depression is not only one of the leading causes of disability in adolescents but is also closely linked to their development in adulthood (27). A longitudinal study tracking adolescents aged 14 to 16 found that individuals with depressive symptoms were 3.99 times more likely to develop depression in adulthood, with a 2.5-fold higher risk for generalized anxiety disorder, and were more likely to face life difficulties such as employment challenges, educational interruption, and relationship issues (28, 29). Adolescent depression is not just an emotional disturbance but can trigger a series of long-term and complex harms. Psychologically, depression leads to persistent self-neglect, emotional regulation disorders, and psychological withdrawal, which exacerbate anxiety, loneliness, helplessness, and suicidal risk (30); behaviorally, adolescents may engage in high-risk behaviors such as aggression, truancy, substance abuse, smoking, and alcohol consumption (31); physiologically, depression has been shown to disrupt adolescents' sleep, endocrine function, and immune systems, increasing the risk of cardiovascular diseases and obesity (32, 33). More critically, depressive symptoms during adolescence have a direct and profound impact on their school social adaptation ability. The emotional lows, cognitive slowness, and lack of motivation caused by depression make it difficult for adolescents to concentrate in class, reducing their academic participation and task completion abilities, thereby threatening their academic achievement (34). Simultaneously, depression severely impairs their interpersonal skills, leading to reduced interactions with teachers, strained peer relationships, and even social trauma such as rejection, bullying, or isolation (35). The Social Functioning Model suggests that depressive symptoms themselves hinder an individual's emotional expression, social judgment, and interaction responses, thereby weakening their ability to perform and connect in social settings (36). Furthermore, Maslow's Hierarchy of Needs theory provides a theoretical foundation: adolescents are in a critical stage of growth and identity formation. If their needs for belonging, love, respect, and self-actualization are unmet in the school environment, it exacerbates their psychological frustration and adaptation difficulties (37). Based on the above review, this study hypothesizes that depression is significantly negatively correlated with adolescents’ school social adaptation (H1).

Under the dominant ideology of “academic achievement above all”, the sleep time of Chinese adolescents has been systematically encroached upon, becoming a structural issue. Early school start times, excessive academic tasks and after-school tutoring, late dismissal times, and late-night internet usage as a form of stress compensation collectively exacerbate their “sleep debt”, leading to the normalization and widespread prevalence of sleep deprivation among adolescents.Sleep quality typically refers to the degree of rest and recovery an individual obtains during sleep, with assessments covering both subjective experiences and objective indicators. Subjective experiences include ease of falling asleep, frequency of night awakenings, morning fatigue, and daytime alertness (38); objective indicators rely on physiological monitoring data, such as sleep duration, sleep latency, sleep efficiency, deep sleep and rapid eye movement (REM) sleep ratios, and nighttime awakenings recorded by polysomnography (PSG) (39). In general, good sleep quality means falling asleep quickly, obtaining adequate sleep, having fewer night awakenings, maintaining a sound sleep structure, and feeling refreshed upon waking (40). According to the National Sleep Foundation's recommendations, adolescents require 8 to 10 h of sleep, while young adults and adults should aim for 7 to 9 h (41). However, in recent years, insufficient sleep and declining sleep quality among adolescents have become a global public health issue. A study of over 270,000 adolescents in the United States found that more than 50% of 15- and 19-year-olds sleep less than 7 h per night (42). Another study involving more than 430,000 middle and high school students reported an average prevalence of sleep disorders at 26% (43). A meta-analysis of 430,000 middle and high school adolescents further confirmed the presence of sleep disorders, with an average prevalence of 26% (44). A sleep quality survey in China also showed that 37.8% of adolescents reported poor sleep quality (45). The factors influencing adolescent sleep quality are complex and diverse. Delayed biological clocks and melatonin secretion during puberty cause adolescents to naturally fall asleep later, and hormonal fluctuations may also interfere with sleep stability and depth. Academic pressure, anxiety, depression, and other emotional issues activate the hypothalamic-pituitary-adrenal (HPA) axis, leading to elevated cortisol levels, which suppress deep sleep and increase night awakenings (46). Poor lifestyle habits, such as prolonged use of electronic devices in the evening, addiction to online gaming, late dinners, smoking, alcohol consumption, or the intake of caffeine and sugary beverages, further delay sleep onset and disrupt sleep structure (47–50). Environmental stressors, such as noise, inappropriate lighting, temperature and humidity, dysfunctional family environments, and lack of social support, also impair sleep quality (51, 52). Among these factors, depression is one of the core psychological variables that influence adolescent sleep quality. Depression not only manifests emotionally as persistent low mood, loss of interest, and rumination but also leads to disruptions in circadian rhythms and abnormal melatonin secretion, making individuals more prone to sleep difficulties, frequent awakenings, early morning awakenings, or excessive daytime sleepiness (32, 33). Under depressive states, negative emotional experiences and chronic psychological stress prolong sleep latency, reduce deep sleep and REM sleep proportions, and increase night awakenings (53). Additionally, depressed individuals often have irregular routines, reduce daytime activities, and engage in poor habits such as increased electronic device use at night, further exacerbating sleep quality decline (54). Poor sleep quality not only weakens immune function and increases the risk of chronic diseases but also severely affects adolescents' cognitive abilities, emotional regulation, and social functioning (55). According to Cognitive Resource Theory, insufficient sleep reduces attention, information processing speed, and emotional recognition abilities, making individuals less able to accurately interpret verbal and non-verbal cues from others in social situations, increasing the risk of inappropriate responses and social conflicts (56). In the school environment, social impairments may lead to peer rejection and a sense of isolation, further weakening school belonging and social support levels (57). Moreover, sleep deprivation also impairs the function of the prefrontal cortex, weakening self-control, planning, and execution abilities, making adolescents more impulsive, anxious, and prone to conflict when facing academic tasks and interpersonal interactions. Over time, declining sleep quality not only lowers academic performance but also directly reduces adolescents' school social adaptation by affecting emotional regulation and interpersonal relationships (58). Based on the above review, this study hypothesizes that sleep quality mediates the relationship between depression and adolescents’ school social adaptation (H2).

As early as 2016, the “Healthy China 2030” planning outline listed “significantly improving the physical health level of adolescents” as an important goal; the “National Fitness Plan (2021–2025)” further made it a mandatory requirement that “middle and primary school students should engage in at least one hour of physical activity on school grounds daily” and increased policy and resource investment in school sports, community venues, and sports guidance services (59). However, in recent years, under the dual influence of the traditional notion of “valuing academics over physical education”, economic downturn, and the real pressure of academic advancement, the physical and mental health issues of Chinese adolescents have become increasingly complex and diverse. Among them, the “Four Small Problems”—myopia, overweight, short stature, and anxiety—have become particularly prominent. These problems often do not exist in isolation, but rather overlap in early onset, high incidence, and comorbidity, forming a systemic health crisis. Several epidemiological surveys indicate that nearly 40% of primary and secondary school students suffer from two or more common health issues simultaneously, with those with comorbid depression and anxiety showing significantly higher risks than those with single conditions. In this context, improving adolescents’ physical and mental health through feasible and cost-effective methods has become a shared focus of both society and academia. Therefore, physical activity has been widely recognized as an important way to enhance adolescents’ health globally (60). Moreover, the academic definition of physical activity has expanded beyond “mere physical expenditure” to emphasize its cultural context and psychological implications. Physical activity can be viewed as a process of movement, action, and performance that takes place within specific socio-cultural and spatial settings, shaped by multiple factors such as individual interests, emotions, motivations, cognitive patterns, and interpersonal relationships. This concept encompasses not only competitive sports and fitness exercises but also diverse forms of movement that carry social interaction and emotional expression meanings, providing a theoretical foundation for understanding the multidimensional benefits of physical activity (61). The World Health Organization (WHO) explicitly recommends that children and adolescents aged 5–17 engage in at least 60 min of moderate to vigorous physical activity daily to prevent chronic diseases and promote mental health and social development (62). However, international monitoring data shows that over 80% of adolescents globally do not meet physical activity levels, and the decline in physical fitness is a widespread problem (63). The United States, through the “Youth Physical Activity Guidelines”, encourages school and community partnerships (64), while the UK's “Chief Medical Officer's Physical Activity Guidelines” specifies daily exercise standards (65), and Australia strengthens sports education and extracurricular participation through the “Sport 2030 National Sports Plan”, all emphasizing the importance of institutional guarantees and social support systems to increase adolescents’ daily activity levels (66). Sustained physical activity offers significant benefits for adolescents’ physical and mental health. Physiologically, regular physical activity enhances cardiovascular endurance, promotes healthy development of bones and muscles, improves metabolic homeostasis and immune function, and lowers cortisol levels by regulating the hypothalamic-pituitary-adrenal (HPA) axis, thereby alleviating excessive physiological arousal responses (67). Additionally, exercise promotes neuroplasticity and the secretion of brain-derived neurotrophic factor (BDNF), optimizing brain structure and function to provide biological support for learning, attention maintenance, and memory processing (68). Psychologically, physical activity helps alleviate depression and anxiety by regulating emotions, buffering stress, and accumulating positive experiences. It also enhances self-efficacy, psychological resilience, and strengthens self-identity and a sense of achievement (69). Cognitively and behaviorally, regular exercise is often associated with healthy lifestyle habits, helping to form regular routines, healthy eating habits, and higher levels of time management and self-discipline (70). In terms of social functioning, physical activity is often accompanied by rich peer interactions and cooperation. High-quality sports friendships provide adolescents with emotional support, trust, and a sense of belonging, significantly enhancing their motivation and persistence, further improving their psychological health and social adaptation with the support of social connections (71). Notably, physical activity also plays a prominent role in optimizing sleep quality—appropriate exercise can shorten sleep latency, increase deep sleep duration, reduce nighttime awakenings, and improve overall sleep structure. This is not only due to the physiological relaxation and circadian rhythm reset brought about by exercise but also closely related to the reduction in anxiety levels, psychological relaxation, and reduced cognitive arousal before sleep (72). For adolescents, regular exercise can also reduce bad behaviors such as electronic device use before bedtime and procrastination in falling asleep, indirectly promoting sleep stability and restoration (73). In summary, the positive effects of physical activity are intertwined across multiple dimensions and mechanisms, collectively promoting a virtuous cycle of physical health, mental health, and social functioning for adolescents, providing solid support for their academic development and social adaptation. Based on this review, this study hypothesizes that physical activity moderates the relationship between depression, sleep quality, and school social adaptation (H3).

**Figure 1:**
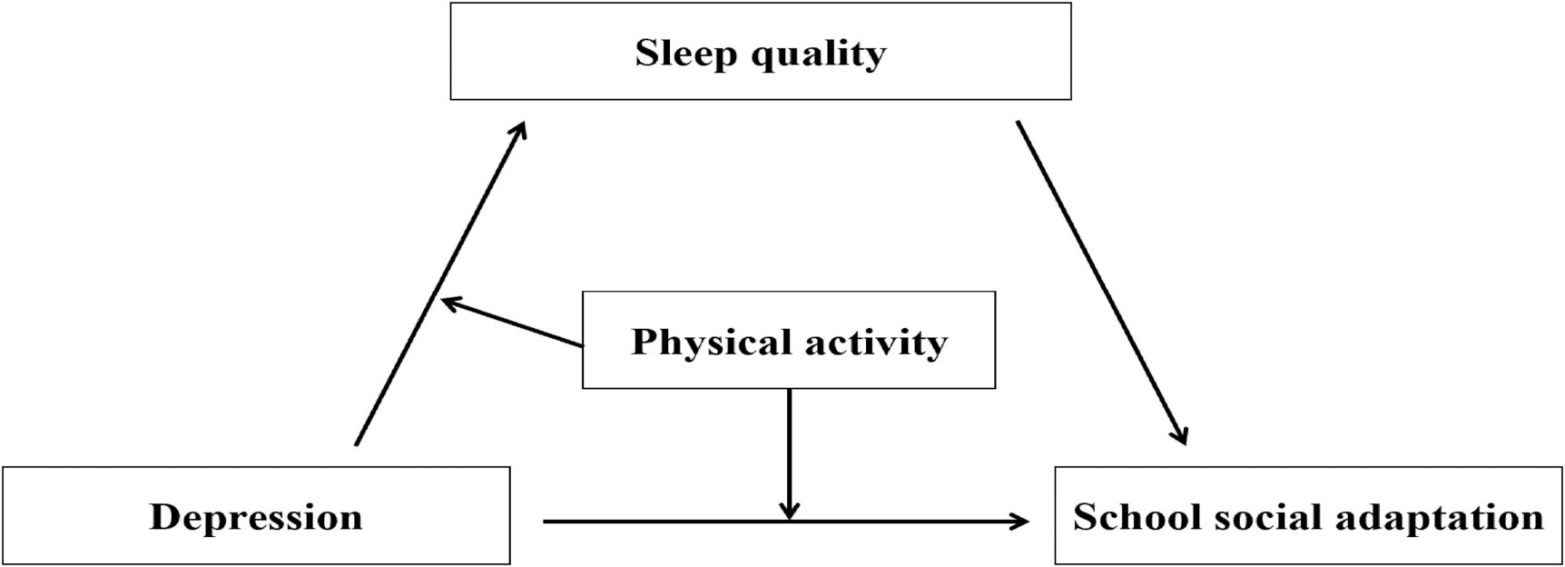
Moderated mediation model.

Based on the above theoretical and empirical research, this study aims to explore the mechanisms underlying the relationships between physical activity, depression, sleep quality, and school social adaptation, and to construct a moderated mediation model based on the existing literature. Specifically, depression, as a persistent negative emotional state, may not only directly weaken adolescents' school social adaptation abilities but also indirectly affect it by impairing sleep quality. Physical activity is considered a potential protective moderating factor, which is expected to buffer the effects along the paths of “depression → sleep quality” and “depression → school social adaptation”. That is, physical activity may reduce the adverse effects of depression on sleep quality and school social adaptation by enhancing individual psychological resilience, emotional regulation, and physiological recovery. Therefore, this study intends to construct a mediation model of “depression → sleep quality → school social adaptation” and introduce physical activity as a moderating variable to reveal its moderating mechanisms in this chain of effects. This will provide a theoretical basis for promoting adolescents' mental health and intervening in social adaptation (see Figure 1).

## 2 Materials and methods

### 2.1 Participants

This study employed a convenience sampling method in June 2025 to recruit 2,500 middle school students from six provinces: Sichuan, Hubei, Guangxi, Gansu, Hebei, and Fujian. Contact and invitations for participation were made through schools and class units. Homeroom teachers or designated teachers assisted in distributing the paper questionnaires and explained the purpose and significance of the study to both students and their parents, inviting them to participate voluntarily. The inclusion criteria for participants were: (1) being a current middle school student; (2) aged between 13 and 18 years; (3) providing informed consent from both the participant and their guardian. The exclusion criteria were: (1) questionnaires with large amounts of missing data or repeated responses; (2) answers showing a high degree of regularity, indicating possible careless responding; (3) excessively short completion time, which could not reflect the participants' true conditions. After screening, a total of 2,354 valid questionnaires were obtained, resulting in an effective response rate of 94.16%. To ensure sample homogeneity, all participants were recruited from regular middle schools, and efforts were made to balance regional distribution, grade levels, and school types to control for potential biases arising from educational background and regional differences. Among the valid samples, 1,098 were male (46.64%) and 1,256 were female (53.36%). The age range was from 13 to 18 years, with an average age of 14.60 ± 1.38 years. Prior to the survey, the research team provided all participants with detailed information about the study's purpose, content, and data confidentiality measures, clearly stating that participation was voluntary and without potential risks, and informed consent was obtained. The questionnaires were distributed on paper through class units, and the research protocol was approved by the ethics review committee of the affiliated institution (Approval No.: JSDX-2023-0071). This study did not collect data on neurodevelopmental diagnoses or substance use; therefore, these factors were not considered as exclusion criteria.

### 2.2 Measurement tools

#### 2.2.1 Depression

Depression levels of the participants were measured using the Patient Health Questionnaire-9 (PHQ-9). This scale consists of 9 items, covering symptoms such as low mood, loss of interest, sleep disturbances, fatigue, appetite changes, self-esteem issues, difficulty concentrating, psychomotor retardation/agitation, and thoughts of self-harm. Each item is rated on a 4-point scale, from 0 (not at all) to 3 (nearly every day), reflecting the frequency of each symptom over the past two weeks. The total score ranges from 0 to 27, with higher scores indicating more severe depressive symptoms. The PHQ-9 has been widely used in both domestic and international studies, demonstrating good reliability and validity in adolescent and university student populations, making it a reliable tool for assessing depressive symptoms (74). In this study, the Cronbach's *α* coefficient of the scale was 0.838.

#### 2.2.2 Sleep hygiene

The Pittsburgh Sleep Quality Index (PSQI) was developed and revised by several authors to measure and assess the sleep quality of university students over the past month (75). The scale consists of 18 self-reported items, which are categorized into 7 components: subjective sleep quality, sleep duration, sleep efficiency, sleep disturbances, use of sleep medications, and daytime dysfunction. Each item is scored from 0 to 3, and the scores of the components are summed to calculate the total PSQI score. A higher score indicates poorer sleep quality. Previous studies have used a PSQI score of ≥8 as the cutoff for poor sleep quality. In this study, the Cronbach's *α* coefficient of the scale was 0.868.

#### 2.2.3 School social adaptation

In this study, school belongingness was measured using the School Belongingness Scale (SBS), developed and validated by Arslan and Duru (76), aimed at assessing middle school students' sense of belonging to school. The SBS consists of 10 items, divided into two dimensions: School Acceptance (5 items) and School Exclusion (5 items). The scale uses a 4-point Likert scale (1 = almost never, 2 = rarely, 3 = sometimes, 4 = almost always), with the items related to exclusion being reverse-scored. After reverse scoring, the total score is calculated. A higher score indicates a stronger sense of belonging to school, while a lower score indicates a higher level of perceived exclusion and alienation. The SBS has demonstrated strong internal and structural reliability coefficients (76). In the original validation study, it showed good reliability and validity, with a Cronbach's α of 0.86 for the total scale, and α coefficients of 0.83 and 0.85 for the School Acceptance and School Exclusion subscales, respectively. Confirmatory factor analysis supported the two-factor structure's applicability in middle school populations (76). In the current study, the Cronbach's α coefficient of the scale was 0.898.

#### 2.2.4 Physical activity

In this study, the Physical Activity Rating Scale (PARS-3) was used to measure the physical activity level of middle school students. This scale assesses the amount of exercise based on three aspects: intensity, duration, and frequency of physical activity. The PARS-3 consists of three subscales: exercise intensity, exercise frequency, and exercise duration, with a total of 3 items. It uses a 5-point Likert scale for scoring. The calculation formula is as follows: Physical Activity Score = Exercise Intensity × (Exercise Duration—1) × Exercise Frequency. The total score ranges from 0 to 100, with higher scores indicating higher levels of physical activity. The Cronbach's α coefficient for PARS-3 in this study was 0.877, demonstrating good internal consistency and reliability (77).

### 2.3 Statistical analysis

Data analysis was conducted using SPSS 27.0. Descriptive statistics and Pearson correlation analysis were first performed on the key variables, followed by the use of Harman's single-factor test to examine common method bias. The results indicated that the first factor accounted for 26.75% of the variance (<40%), suggesting that no significant common method bias was present. Subsequently, the SPSS PROCESS macro (Models 4 and 8) was used to test the moderated mediation model, with 5,000 bootstrap samples (95% confidence interval) to examine the moderating role of physical activity in the pathway through which depression affects school social adaptation via sleep quality. All statistical significance levels were set at *p* < 0.05.

## 3 Results

### 3.1 Common method bias test

To assess the impact of common method bias, Harman's single-factor test was conducted in this study. The results revealed that, without performing principal component factor rotation, three factors had eigenvalues greater than 1. The variance explained by the first factor was 26.746%, which is below the 40% threshold. Therefore, it can be concluded that no significant common method bias was detected in this study.

### 3.2 Correlation analysis

The results in Table 1 indicate that depression is significantly negatively correlated with both sleep quality and school social adaptation. Additionally, physical activity shows strong correlations with depression, sleep quality, and school social adaptation.

**Table 1:** Correlation analysis.

Variables	M	SD	1	2	3	4
1 Depression	0.630	0.504	–			
2 Sleep quality	1.206	0.528	−0.309***	–		
3 School social adaptation	2.878	0.635	−0.410***	0.326***	–	
4 Physical activity	2.463	0.773	0.264***	−0.181***	−0.204***	–

*** *p* < 0.001 (highly significant).

### 3.3 Mediation model test

**Figure 2:**
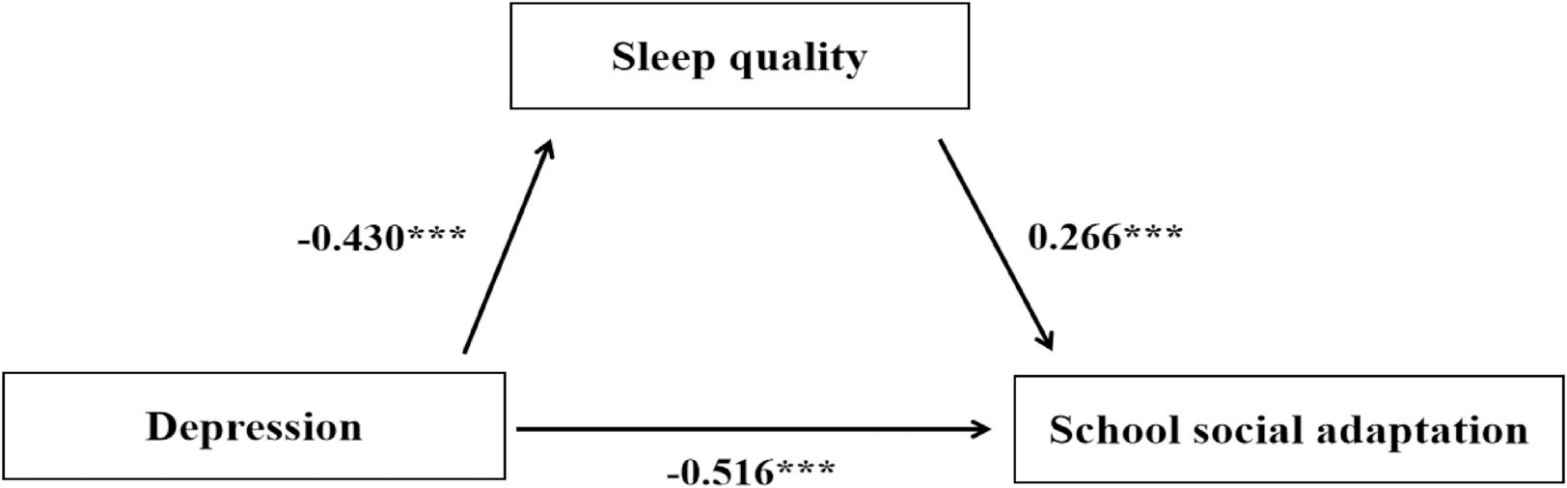
Mediation model diagram.

According to the results in Table 2 and Figure 2, depression significantly negatively predicted school social adaptation (*β* = −0.516, *p* < 0.001). This predictive effect remained significant even after including sleep quality as a mediating variable. Additionally, depression had a significant negative effect on sleep quality, while sleep quality significantly positively predicted school social adaptation. Further analysis indicated that sleep quality played a significant mediating role in the relationship between depression and school social adaptation. The specific path effects are detailed in Table 3.

**Figure 3:**
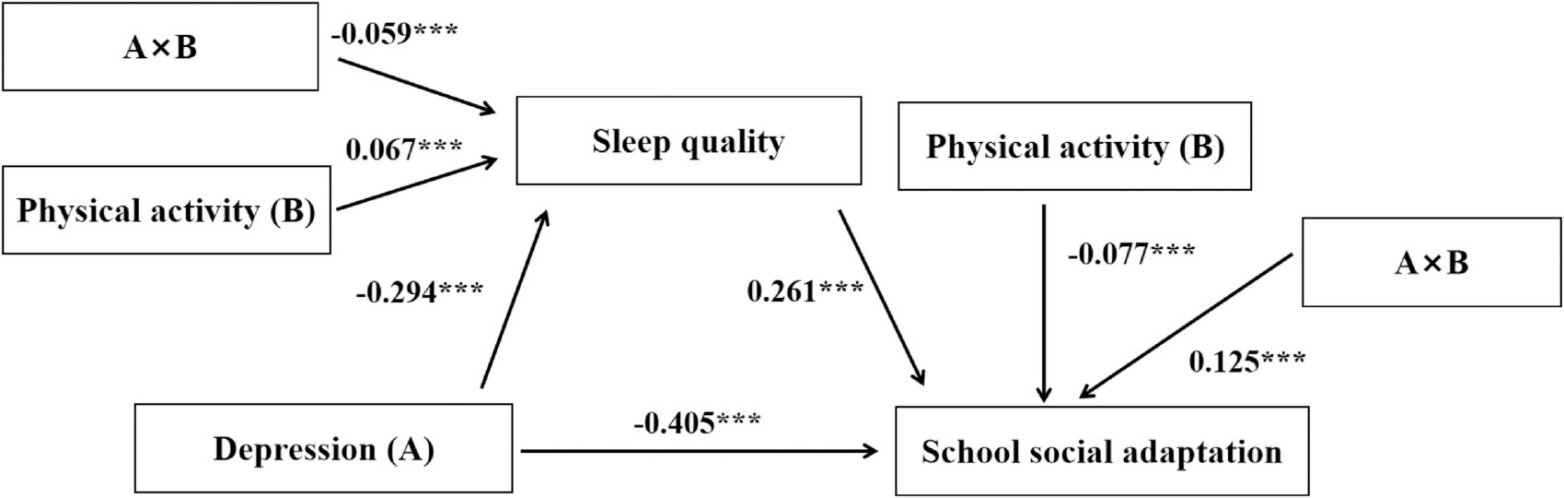
Moderated mediation model (***: *p* < 0.001).

**Table 2:** Mediation model test.

Outcome variables	Predictor variables	*β*	SE	t	*R*²	F
School social adaptation	Depression	−0.516	0.024	−21.784***	0.168***	474.540***
School social adaptation	Depression	−0.430	0.024	−17.744***	0.212***	316.372***
	Sleep quality	0.266	0.023	11.481***		

*** *p* < 0.001 (highly significant).

**Table 3:** Mediation model path analysis.

Intermediate path	Effect size	SE	Bootstrap 95% CI	Proportion of mediating effect
Total effect	−0.516	0.024	−0.470, −0.410	
Direct effect	−0.430	0.024	−0.383, −0.342	83.33%
Total indirect effect	−0.086	0.10	−0.106, −0.068	16.67%

### 3.4 Moderated mediation model test

The results of the moderated mediation model test (Table 4, Figure 3) indicated that indicated that physical activity moderated the pathway through which depression affects school social adaptation. Specifically, physical activity significantly moderated the effect of depression on sleep quality (A × B interaction: *β* = −0.059, *p* < 0.001), and also moderated the direct effect of depression on school social adaptation (A × B interaction: *β* = 0.125, *p* < 0.001).

**Table 4:** Has moderated mediation model tests.

Outcome variable	Predictor variable	β	SE	t	*R*²	F
Sleep quality	Depression (A)	−0.294	0.021	−13.887***	0.108	94.939***
	Physical activity (B)	−0.067	0.014	−4.817***		
	A*B	−0.059	0.024	−2.448***		
School social adaptation	Depression (A)	−0.405	0.025	16.432***	0.225	170.571***
	Sleep quality	0.261	0.023	11.271***		
	Physical activity (B)	−0.077	0.016	−4.873***		
	A*B	0.125	0.027	4.658***		

* *p* < 0.05.

*** *p* < 0.001.

**Table 5:** Predictive effects of different physical activity levels.

Physical activity Levels	Effect size	SE	t	Lower limit 95% CI	Upper limit 95% CI
Low	−0.249	0.028	−8.853***	−0.304	−0.194
Medium	−0.294	0.021	−13.887***	−0.336	−0.252
High	−0.339	0.028	−12.078***	−0.394	−0.284

*** *p* < 0.001 (highly significant).

**Figure 4:**
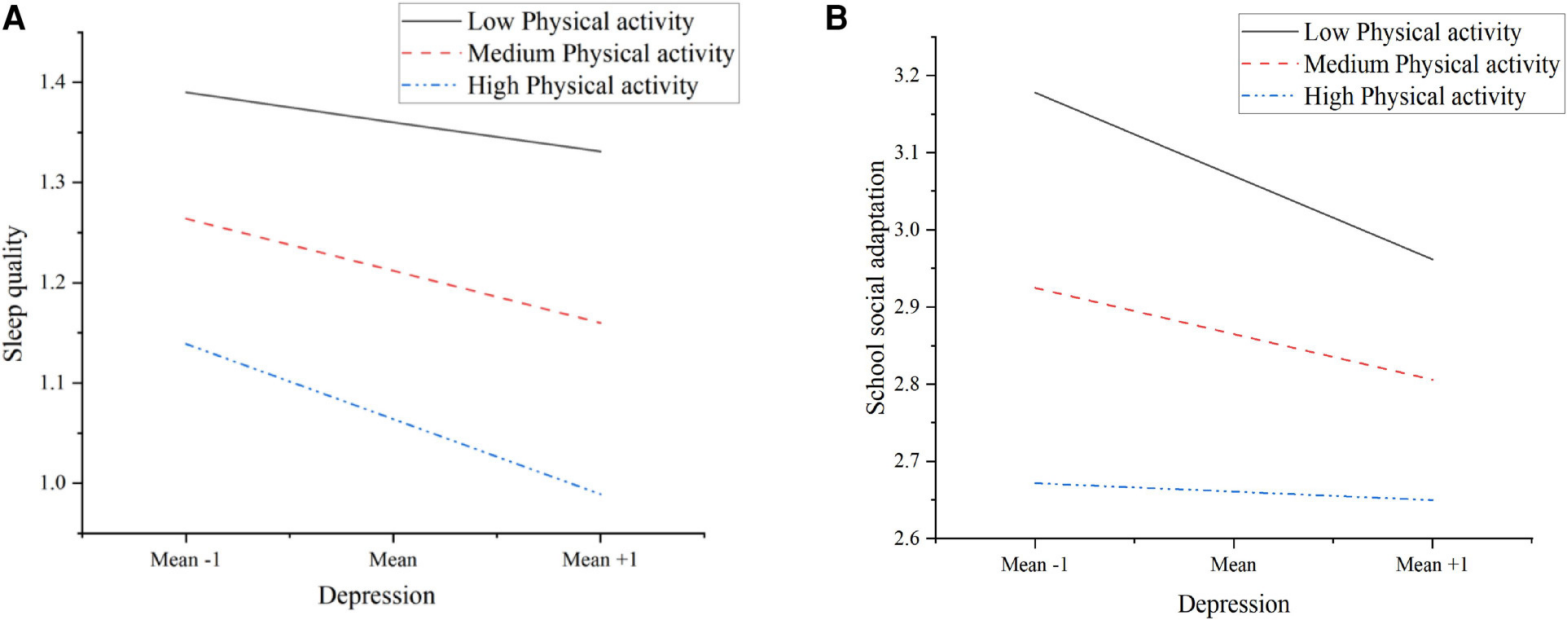
Simple slope analysis. **(A)** The moderating effect of physical activity on the relationship between depression and sleep quality. **(B)** The moderating effect of physical activity on the relationship between depression and school social adaptation.

The results of the simple slope analysis (Figure 4, Table 5) further revealed the protective role of physical activity. As the level of physical activity increased, the negative predictive effect of depression on sleep quality gradually weakened. Similarly, in the direct pathway of depression predicting school social adaptation, physical activity also demonstrated a similar protective pattern. The positive interaction term (*β* = 0.125) suggests that as physical activity levels increase, the direct negative impact of depression on school social adaptation decreases.

These findings collectively highlight the protective role of physical activity in the mechanism through which depression affects school social adaptation via sleep quality, providing empirical evidence for improving adolescent mental health and school adaptation by promoting physical activity.

## 4 Discussion

The results of this study confirm hypothesis H1. In the context of China's educational ecosystem, the negative impact of depression on school social adaptation is amplified due to its conflict with the dominant academic performance norms. Depressed students exhibit signs of distracted attention, reduced social interactions, and marginal participation in group activities (such as passively responding during group discussions or avoiding collaboration in physical education classes), which not only weakens their academic engagement and emotional support acquisition but also poses a potential threat to their social identity. In an environment that places a high value on visible participation and conformity, such avoidance-based self-presentation strategies significantly damage an individual's “qualified student” identity in the eyes of peers and teachers. This, in turn, accelerates the process of psychological withdrawal and social alienation, ultimately deeply restricting their school adaptation levels (78). From a theoretical perspective, social withdrawal theory suggests that emotional distress and negative self-cognition drive individuals to reduce social behaviors to avoid the risk of rejection or conflict. Depressed adolescents, due to their low self-esteem and heightened sensitivity to social feedback, are more likely to withdraw from social situations, thus becoming trapped in a “withdrawal-isolation-adaptation impairment” vicious cycle (78). In terms of physiological mechanisms, depression is closely associated with dysfunction of the hypothalamic-pituitary-adrenal (HPA) axis, which is characterized by prolonged elevated cortisol levels and excessive activation of the autonomic nervous system. This chronic stress state not only weakens attention, executive functions, and memory processing but also leads to abnormalities in neurotransmitters such as serotonin and dopamine, as well as mild neuroinflammation. These physiological disruptions further impair cognitive and emotional regulation, thereby reducing learning efficiency and decreasing the flexibility of social responses (79). Even when depressed students have a subjective desire to participate in class and group activities, they may struggle to keep up with the pace of the class due to mental fatigue, lack of focus, and low physical energy (80). In team-based situations, they often become marginalized by their peers due to slow reactions or lack of initiative, which unintentionally diminishes their social participation and further exacerbates the decline in their school social adaptation (81). From a developmental psychology perspective, adolescence is a critical period for constructing social identity and role recognition. The social withdrawal and reduced participation induced by depression can undermine the formation of a positive social identity and restrict the development of social skills. This negative trajectory may persist into adulthood, affecting interpersonal relationships, career adaptation, and mental health (82). In inclusive educational environments, the impact of depression on school social adaptation is relatively mild. However, in East Asian regions like China, where there is significant pressure related to academic performance, this negative effect is more pronounced. This effect may be further amplified by the combined pressures of academic achievement, teacher expectations, and parental demands (83).In conclusion, within the cultural context of exam pressure and societal expectations, depression not only influences adolescents’ school social adaptation through psychological mechanisms such as withdrawal and avoidance, but it also has a comprehensive impact on their adaptation due to HPA axis dysfunction, neurotransmitter abnormalities, and other related factors (H1).

Chinese adolescents are immersed in an “evaluation-driven” educational ecosystem, jointly constructed by a system focused on selection and streamlining, and a peer environment rife with high social comparison pressures. In this ecosystem, individuals are continuously exposed to achievement anxiety, which becomes a significant breeding ground for depressive emotions. Once they fall into depression, the typical rumination and emotional dysregulation become compounded by external institutional time pressures (such as prolonged school hours and after-school tutoring), creating a dual dilemma of internal and external depletion. This dilemma leads to a sleep process that should be relaxing becoming filled with both cognitive and emotional interference, significantly disrupting the initiation and maintenance mechanisms of sleep, ultimately causing a sharp decline in sleep quality (84). The results of this study can be further elucidated within this context: in the current educational environment where the pressure of academic advancement is heavy, depression not only directly weakens adolescents’ interpersonal skills but, more importantly, severely impairs sleep quality, which in turn indirectly and profoundly impacts their overall school social adaptation levels. Research shows that depression often accompanies imbalances in the regulation of the sleep-wake system, including disrupted melatonin secretion rhythms, reduced deep sleep proportions, and increased night-time awakenings. These sleep structure abnormalities not only directly impair the restorative functions of sleep but also damage memory processing, emotional stability, and behavioral control (85). For adolescents already in a depressive state, the risk of sleep disruption is particularly pronounced in the Chinese educational environment. The long-standing focus on academic exams and grade rankings forces many students to sacrifice sleep to manage their academic workload. Depression further reduces sleep efficiency, delays sleep onset, and increases nighttime awakenings, creating a cumulative effect of dual pressure that further exacerbates sleep quality (84). On a psychological level, depression intensifies ruminative thinking and negative self-evaluations, making it difficult for individuals to relax before sleep. This leads to the development of poor habits such as staying up late, prolonged use of electronic devices, and overeating, which further disrupts sleep quality. The consequences of poor sleep quality extend beyond simple fatigue; they can trigger a chain reaction in multiple cognitive, emotional, and behavioral dimensions (86). According to cognitive resource theory, sleep deprivation reduces the cognitive resources available to an individual, impairing their ability to accurately interpret nonverbal cues and recognize others' emotions in social situations. This social perception bias can lead to miscommunication, awkward interactions, and even peer exclusion (87). Building upon this, the emotional regulation process model suggests that individuals with poor sleep are less likely to use effective strategies for emotional regulation early in the emotional generation process. Instead, they tend to rely on less effective strategies such as rumination or suppression, which aggravates emotional distress and increases the risk of interpersonal conflict (88). In the school environment, this means that adolescents with poor sleep quality are often slow to respond in classroom discussions, lack cooperation, and show reduced patience and coordination in group tasks, which weakens peer relationships and social status (89). Furthermore, prolonged sleep deprivation lowers neural activation levels in relevant brain regions, leading to increased impulsivity, hindered planning, and decreased executive function in tasks requiring self-regulation. This weakening of self-control not only leads to distracted attention in class and delayed homework completion but may also increase conflict behavior and violations of school rules, further obstructing the development of school social adaptation (90). Existing research has shown that the negative impact of sleep deprivation on the prefrontal cortex function and self-regulation abilities in adolescents can persist for years (91). Neuroimaging studies have similarly found that adolescents with poor sleep quality show significantly reduced prefrontal cortex activation in tasks related to emotional and behavioral regulation (92). In summary, sleep quality plays a crucial role in emotional regulation, behavioral optimization, and the maintenance of neural function. It serves as an important mediating variable in the relationship between depression and school social adaptation, as well as a protective psychological resource (H2).

In the current educational reality, physical education scores are not included in the total score for the Gaokao (college entrance exam), and even in the Zhongkao (high school entrance exam), its limited score is often obtained through last-minute cramming. This has led to the widespread phenomenon of “physical education classes being occupied by core subjects”. Under the dual pressures of the societal notion that “academics are superior to physical education” and parents' expectations for their children to excel, adolescents’ time is highly concentrated on the “academic main track”. Against this backdrop, this study found that physical activity plays an active moderating role in both the “depression—sleep quality” and “depression—school adaptation” pathways. First, in the “depression → sleep quality” pathway, physical activity buffers the negative impact of depression mainly through cognitive emotional regulation and physiological coordination. On a psychological level, ruminative thinking and emotional disturbances caused by depression are the primary disruptors of sleep. The exercise process can effectively release stress, burn excess energy, and interrupt rumination, making it easier for individuals to enter a relaxed state before sleep. Moreover, the sense of accomplishment and self-efficacy gained from regular exercise also helps to lower emotional vigilance, reducing nighttime awakenings triggered by emotional fluctuations (93). On a physiological level, regular and moderate aerobic or resistance exercises can increase slow-wave sleep proportion, extend deep sleep duration, reduce nighttime awakenings, and shorten sleep latency while stabilizing nighttime rhythms by regulating body temperature reduction and balancing the autonomic nervous system (94). Additionally, the moderate fatigue induced by exercise promotes the secretion of *γ*-aminobutyric acid, serotonin, and melatonin in the brain, which directly participate in the initiation and maintenance of sleep (95). In the daily context of Chinese adolescents, even during periods of high academic pressure, moderate extracurricular physical activities (such as jogging, ball games, swimming) can still provide relatively stable sleep protection, thus buffering the dual depletion of physiological and psychological stress. Secondly, in the “depression → school social adaptation” pathway, physical activity works by providing alternative sources of value and opportunities for social reintegration. Previous studies have also found that regular exercise can enhance prefrontal cortex function, improve executive control and cognitive flexibility, making students more focused and efficient in classroom and team tasks (96). On a psychological level, when adolescents become trapped in self-doubt due to depression amidst standardized academic competition, physical activity offers a reference frame based on individual progress. Every breakthrough in endurance or skill mastery serves as recognition of one's own abilities, and this sense of achievement, obtained through direct physical experience, can effectively deconstruct the negative self-perception built from academic setbacks. More importantly, this achievement does not rely on social comparison with others but is rooted in a vertical comparison of personal growth, thus providing a stable and controllable source of self-worth for depressed individuals, reshaping their motivation and self-identity (97). On a social connection level, physical activity constructs a non-verbal interaction domain. Depression is often accompanied by social anxiety and difficulty in expression, while interaction in collective sports (such as a tacit tactical collaboration or an encouraging high-five) relies on pre-verbal physical dialogue and empathy. This interaction mode significantly reduces social anxiety caused by excessive focus on others' evaluations. In the process of pursuing a common team goal, individuals experience interpersonal affirmation based on cooperation rather than competition, and this experience can effectively repair the relationship network damaged by social avoidance and rebuild a sense of belonging and emotional security within the collective identity (98, 99). This social connection based on bodily co-presence provides depressed adolescents with an entry point to re-engage in social activities. In summary, the above evidence supports the hypothesis of this study that physical activity plays a moderating role between depression and sleep quality, and between sleep quality and school social adaptation (H3).

This study investigates the underlying mechanisms linking depression, sleep quality, and school social adaptation, with physical activity as a moderating factor, and proposes a potential path model based on these relationships. However, several limitations need to be addressed. The primary limitation lies in its cross-sectional design. Although we constructed the “depression → sleep quality → school adaptation” model based on theory and found significant statistical associations, cross-sectional data cannot provide conclusive evidence for causal directions between variables. For example, there may be reverse causality (e.g., school adaptation difficulties exacerbating depressive symptoms) or dynamic bidirectional influences. These complex causal chains could not be tested in the current design. Secondly, the data primarily come from self-reported surveys. While the measurement tools used have high reliability and validity and are widely applied both domestically and internationally, self-reported data are inevitably subject to recall bias, social desirability effects, and individual differences in subjective interpretation. Thirdly, regarding model construction and validation, while we validated the moderating effect of physical activity in the model, the exploration of this effect remains relatively broad. Different types of physical activity (e.g., open-skill vs. closed-skill sports), different exercise contexts (e.g., self-selected vs. forced participation), social interaction features in exercise (e.g., cooperation vs. competition), as well as the combined effects of exercise intensity and frequency, may all exert influences through different psychological and physiological pathways. These finer moderating mechanisms warrant further exploration. To address the above limitations, future research could delve deeper into the following areas: First, in terms of research design, longitudinal tracking with multiple time points could be employed to examine causal relationships between variables using cross-lagged panel models or latent growth models to explore the developmental trajectories and interrelations of the variables. For instance, assessments could be conducted each semester and followed for 2–3 years to better reveal dynamic interactions between variables. Second, in terms of data collection methods, multi-method and multi-source data integration strategies could be advanced. In addition to self-reported questionnaires, wearable devices such as accelerometers and heart rate variability monitors could be used for objective monitoring of physical activity levels and sleep patterns. Salivary cortisol, heart rate variability, and other physiological indicators could be collected to assess stress responses. Meanwhile, multi-source data from teacher evaluations, peer nominations, and parent reports could be integrated to build a more comprehensive and objective evidence chain. Third, in terms of statistical analysis methods, model validation and result presentation could be further optimized. When analyzing using structural equation modeling, model path diagrams should provide complete information, including standardized path coefficients, confidence intervals, and model fit indices. When presenting moderating effects, besides reporting the significance of interaction terms, simple slope analysis and graphical methods could be used to visually show the differences in how depression affects the outcome variables at different levels of physical activity. For presenting mediation effects, confidence intervals for both specific indirect effects and total effects should be reported to enhance the interpretability of the results. Future research could also consider conducting randomized controlled trials on exercise interventions to systematically examine the causal effects of different types, intensities, and frequencies of physical activity on sleep quality and school adaptation in adolescents with depression, exploring the underlying psychological and physiological mechanisms. This would provide empirical evidence for developing targeted exercise intervention programs. Advancing these research directions will help build a more comprehensive theoretical model and provide more targeted practical guidance for promoting adolescent mental health and school adaptation.

## 5 Conclusion

This study systematically explores the mechanisms through which depression affects adolescents' school social adaptation, clarifies the mediating role of sleep quality, and verifies the moderating effects of physical activity in the “depression—sleep quality” and “depression—school social adaptation” pathways. Based on the findings, the study recommends constructing a comprehensive intervention system with multi-party collaboration. At the school level, the following systematic measures should be implemented: First, strictly enforce the “two hours of exercise per day” policy, create high-quality physical education curricula, organize school-wide and grade-wide sports competitions, and implement a “15-minute break exercise” system to effectively extend break activity time and enrich after-school physical activity programs. Second, establish a sound sleep health management system, include students' sleep conditions in the physical health monitoring and educational quality evaluation systems, scientifically arrange school start and end times, prohibit early arrivals, and prevent behaviors that encroach upon sleep time such as extended learning sessions. Additionally, provide sleep health education through seminars and class meetings. Third, establish a “class teacher-psychological counselor-psychiatrist” three-level coordinated psychological health service system to promote early identification and professional intervention of students' psychological problems. Fourth, strengthen the parent-school cooperation mechanism, organize at least two family education guidance activities per semester, focus on psychological health, guide parents to pay attention to their children's physical and mental development, and actively create a family-oriented sports atmosphere. Fifth, actively integrate community resources to co-build a psychological service platform, support professional services for children's and adolescents' mental health, and form a school-community collaborative education network. At the societal level, it is urgent to promote the reform of the education evaluation system, break the single evaluation standard of “exam results only”, and create a diverse talent development environment through media promotion and community education. At the same time, students' physical and mental health levels, social adaptation ability, and innovative literacy should be included as core indicators in the education quality evaluation system, fundamentally improving the incentive mechanisms and educational orientation of basic education.

## Data Availability

The datasets generated and/or analysed during the current study are not publicly available due to our experimental team's policy but are available from the first author on reasonable request. Requests to access the datasets should be directed to Chuan Chen, 1377943484@qq.com.
